# An Experimental Study of Auxetic Tubular Structures

**DOI:** 10.3390/ma15155245

**Published:** 2022-07-29

**Authors:** Julian Plewa, Małgorzata Płońska, Kamil Feliksik

**Affiliations:** Faculty of Science and Technology, Institute of Materials Engineering, University of Silesia in Katowice, 75 Pułku Piechoty Str. 1a, 41500 Chorzów, Poland; kamil.feliksik@us.edu.pl

**Keywords:** auxetic materials, negative Poisson’s ratio, rotating squares, planar and tubular structures

## Abstract

Auxetic tubular structures are widely known structures, characterized by a negative Poisson’s ratio upon stretching and deformation in the axial and transverse directions, which have numerous application possibilities. In this paper, tubular structures were realized by rolling up planar auxetic structures and using rigid square frames as unit cells. Planar and tubular structures were built from square frames that were 3D printed with plastic or laser-cut from metal. The changes in linear dimensions of the studied structures were based on a hinge mechanism, the functioning of which was experimentally verified on different solutions leading to square unit cells. To connect the square frames of the structure, an innovative solution was used in the form of rotation axes on their surface at a preset distance from the edge of the square frame. The geometric parameter thus introduced was used to determine the relative change in the size of the structure when stretched (i.e., when moving from the closed to the open position).

## 1. Introduction

Auxetic structures belong to a group of metamaterials which include numerous groups of artificial structures with properties not found in nature. They are the most intriguing kind of mechanical metamaterials in that they have been invented on a counterintuitive basis. The beginning of scientific recognition of metamaterials is due to physicists who found no contradiction in the possibility of negative material coefficients in their defining equations.

Beginning with Veselago [1], a Ukrainian researcher who suggested the possibility of simultaneous negative magnetic permeability and electric permittivity, through Pendry [2], who analyzed metamaterials with negative refractive index and magnetic permeability, Almgren [3], who presented models of the mechanical structure with negative Poisson’s ratio, to Lakes [4], who pointed out the negative stiffness of metamaterials, these types of materials are rapidly being developed.

The great popularity of mechanical metamaterials exhibiting negative Poisson’s ratio, i.e., auxetics, was started by Wojciechowski [5,6] with a model of chiral structures, Lakes [4] with polyurethane foams, and Evans with microporous materials, among others [7].

When it comes to nomenclature, the word ‘auxetic’ comes from the Greek word auxetikos (αὐξητικός) which means “that which tends to grow” and was proposed by Evans et al. in 1991 [8]. In practice, it applies to those materials that increase their transverse dimension under axial tensile stress, resulting in a negative Poisson’s ratio.

In addition to chiral structures, a significant number of auxetic structures have emerged in which the unit cells are specific man-made geometric constructions. These include variants of the complex cellular structures of two-dimensional concave and convex honeycombs [9]. This type of unit cell may have its origin in nature, but most of them are a product of human invention. Nevertheless, examples of auxetics of natural origin can also be found, e.g., in cristobalite (polymorphic silicones) [10,11,12], in some intermetallic phases, e.g., Cu_68.6_Al_27.6_Ni_3.8_ [13], zeolites [14], in quartz under reduced hydrostatic pressure [12], and in biological tissues such as cancellous bone [15], tendons [16], and the skin of some animals [17,18].

**Figure 1:**
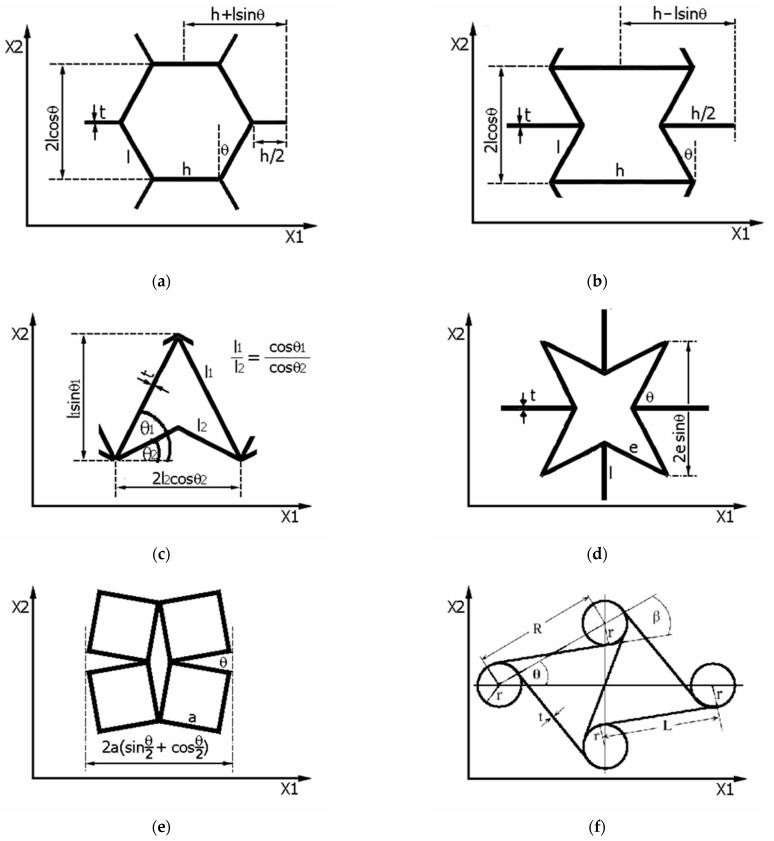
Commonly recognized unit cell models: convex and concave honeycomb unit cells (**a**,**b**), arrowhead (**c**), star (**d**), rotating squares (**e**), and chiral cells (**f**).

Figure 1 shows diagrams of unit cells, from which regular auxetic structures are formed by combining them. Classical designations and relationships for unit cells are shown in commonly recognized models of convex and concave honeycomb, arrowhead, star, rotating squares, and chiral cells.

The first two types of unit cells (Figure 1a,b) have their origin in the work of Gibson et al. (1982) [9], Almgren (1985) [3] and Kolpakov (1985) [19], while others were discovered by Larsen et al. [20], Theocaris et al. (1997) [21], Grima and Evans (2000) [22], and Wojciechowski (1987) [5,6]. Currently, we can already name about 32 types of elementary unit cells, with auxetic behavior confirmed for about 20 of them [23].

**Figure 2:**
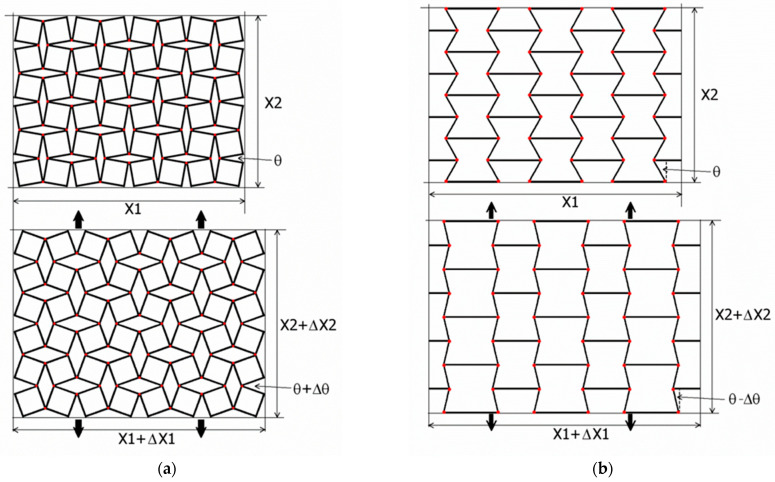
Models of auxetic structures built from unit cells in the form of rigid squares (**a**) and re-entrant—‘bow tie’ cells (**b**) with hinges marked by red dots.

Auxetic structures are formed by connecting unit cells, with these connections in theoretical considerations being points of contact, while in physical models, they are a type of a hinge. Flexible hinges (shown as the thickened ends of the elementary cells—Figure 2) at the cell contact points allow the cell to deform when stress is applied and to revert to its initial position when the stress goes away. Figure 2 shows the behavior of auxetic structures formed from two types of unit cells, from rigid squares and from re-entrant cells subjected to the stretching of the structure.

As is well known, auxetic behavior is induced by elastic deformation, which, in addition to hinging, may also involve further two deformation mechanisms: bending and stretching/compression of unit cells. When there is a hinge mechanism involved, the transition from *closed* to *open* position is more a result of the Poisson’s ratio, which corresponds mainly to an application where the initial and final states of the structure are relevant.

Although the hinges marked in the diagrams allow the cells to rotate and deform, this can only occur within a very limited range, which is expressed by a small change in the angle Δθ (Figure 1).

Figure 2 shows the dimensional changes of the structure upon stretching. In the case of a structure based on rigid squares (Figure 2a), as a result of stretching, the dimensions of the structure increase and the theta angle changes. This structure can theoretically be stretched until the theta angle reaches 90°.

In the case of a structure assembled from re-entrant unit cells (bow ties), the increase in linear size is accompanied by a decrease of the theta angle so that the theoretical theta angle reaches zero 0° at maximum stretch. In this case, auxetic behavior occurs mainly due to the movement (hinging and bending) of the transverse ribs, although re-entrant cells can also deform as a individually (whole). It should be noted that the discussed expansion of the dimensions of the structure takes place in particular through the use of hinged connections. For rigid-square structures, these hinges are present only where the vertices of the squares meet, whereas for re-entrant (bow-tie) structures, hinges are present at all joints.

Generally, in hinging, the cell walls are rigid in both the axial and transverse directions, and the strain produced by the tensile force is expressed by a change in theta angle, but for infinitesimally small strains—which is limited by the elastic properties of the hinge material. It can be added that the variation of the theta angle, together with other geometric elements of unit cells (including the thickness t), forms the basis of simulation analyses. Especially notable among these numerous studies are those of Gibson and Ashby [24] and Masters and Evans [25], in which the mechanical properties of auxetic structures were determined in global form considering the effect of load on displacement. These and similar virtual procedures are now a major part of all research on auxetics [26,27,28,29,30,31,32,33].

Considering actual auxetic designs, it is necessary to solve the issue of hinges. This is due to the fact that these areas absorb most of the stress concentration in the system while allowing the unit cells to rotate. When critical stress values are reached, which depend on the elastic properties of the unit cell material, it leads to hinge failure. Functioning of the hinges occurs only to a limited extent, and thus the auxeticity of the structure may be compromised. Perfectly flexible hinges are utilized in theory, but actual physical implementations find such perfection difficult, or rather impossible, to achieve.

The motivation of this work is to find solutions to the problem of imperfect hinges connecting unit cells and allowing them to rotate and move. In the present work, a new innovative solution was applied to create robust unit cell connections by introducing a rotation axis on the surface of rotating elements forming unit cells. This solution has been successfully tested for auxetic constructions based on rotating squares [34].

The experimental studies consisted of an attempt to verify the performance of metamaterial structures built of square unit cells, both by geometrical analysis and by generating physical models, taking into account the influence of the cell shape and the materials used for the unit cells.

## 2. The Experiments

In the experimental part, the modified auxetic constructions based on square unit cells were analyzed, first based on geometrical considerations of the construction and then step-by-step implementation of it in demonstrations. By choosing this method of analysis, the topologies and functioning of the structures were tested.

### 2.1. Two-Dimensional Auxetic Constructions from Rigid Squares

In the present work, the rotating square structures invented by Grima and Evans [22] are used, formed from rigid solid squares that have been modified by introducing the axis of rotation on the surface of the squares near their corners. This produces stable auxetic structures that can function reliably without the danger of damage or destruction.

By solving the problem of stable connections of rigid squares that retain their rotational motion by introducing the axis of rotation at the corners of the squares, an auxetic structure exhibiting large values of longitudinal and transverse linear expansion was obtained [34]. Thus, using just the hinge mechanism for rigid squares allowed us to obtain auxetic structures exhibiting a negative Poisson’s ratio (NPR) of −1. By connecting the square unit cells to the rotation axes, the resistance to rotation of the squares is almost completely eliminated, and large changes in the theta angle can be achieved.

The expansion of structures built from such connected rigid square unit cells does not depend on the properties of the material, so there is no functional relationship between the theta angle and Poisson’s ratio (NPR). The Poisson’s ratio, or more precisely, the quotient of the relative change in the transverse dimensions and the axial dimensions, will, in this case, only be related to the change in the linear dimensions. In each case, for such structures it reaches −1 and remains constant regardless of the degree of opening of the structure and regardless of its size.

**Figure 3:**
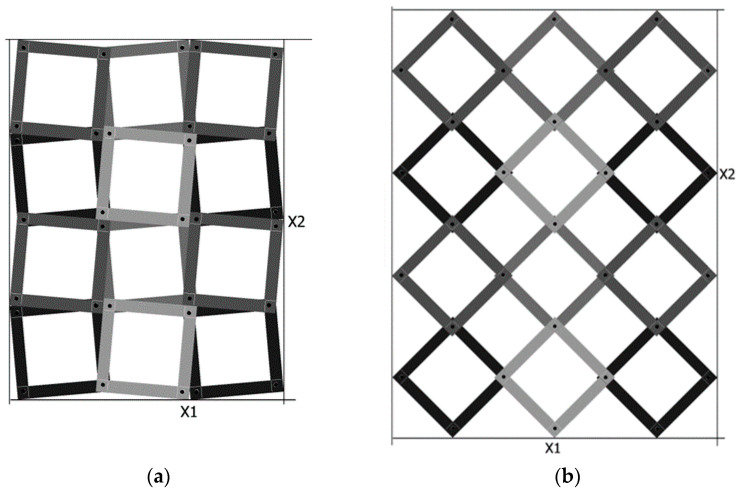
Auxetic structures made of rigid rotating square frames in the closed position (**a**) and in the open position (**b**).

A new proposal based on rotating squares are square frames combined into a structure that exhibits auxetic properties (Figure 3). The geometric structure modelled here is an example of a lattice structure with varying clearance. This design is limited to a specific geometry and to a specific stiffness of the unit cell material.

By stretching the presented structure in the longitudinal direction, it also expands in the transverse direction. This is the result of the interaction between rigid square frames connected by axes of rotation. It should be noted that the expansion of the structure occurs not only as a result of rotational motion but also as a result of the movement of the frames (a rotation-translation mechanism).

**Figure 4:**
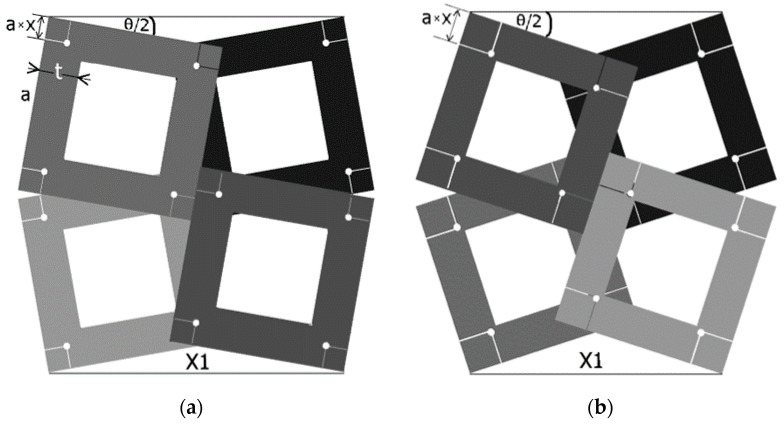
Structure of 2 × 2 rotating square frames in the closed position, with the geometric parameters x and θ marked for two different values of the x parameter, (where (**a**) x = 0, and (**b**) x = 0.25), and the θ angle.

The position of the axis of rotation on the surface of the square frame was defined using the introduced geometric parameter x, which, with an outer dimension of the frame a, defines an edge distance of a × x. Figure 4a shows the structure of the rotating square frames in the closed position.

From the geometrical analysis of the presented structure, the following relationship [34] arises:

(1) $$\tan\frac{\theta }{2}=\frac{1+\sqrt{1-4x}}{1-\sqrt{1-4x}}$$

This relationship allows the square cells to be specifically arranged when the value of the parameter x is chosen, though with it being between 0 and 0.25. As the parameter x increases, the θ angle increases steeply. For x = 0, the square cells are connected at the corner contact point (practically infeasible), and for x = 0.25, the structure becomes locked as a result of the square cells interlocking with each other. Figure 4 also shows that as the parameter x increases, the size of the structure shortens.

### 2.2. Auxetic Tubular Structures

Tubular, pipelike structures are among the mechanical metamaterials particularly sought in engineering applications. For this reason, numerous proposals can be found in the literature, one group of which concerns medical applications [35,36,37,38,39,40,41].

**Figure 5:**
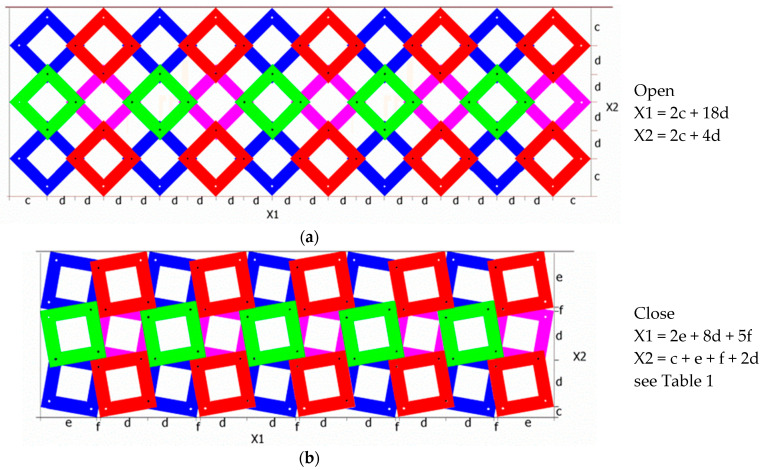
A 3 × 10 planar auxetic structure of rigid square frames in open (**a**) and closed (**b**) positions, along with the method of calculating its linear size.

An auxetic planar (2D) structure constructed from rotating square frames connected by axes of rotation on the surface of the squares can be rolled up and connected to form an openwork tube. Such a tubular structure can also be shown to have auxetic properties. Figure 5 first shows the initial 3 × 10 planar structure in the open position and in the closed position.

The determined values of size X1 and X2 can be used to calculate the amount of expansion due to the transition from the closed to the open position. Figure 5 shows the individual segments, the sum of which yields the values X1 and X2. The lengths of the individual segments can be found by considering the geometric relationships of the structure made of squares. The values of X1 and X2 depend on the number of segments, as well as on the parameter a and the angle θ.

The parameters used in the formulae in Table 1 are the following: a—the length of the square side of the unit cell, and θ—the inner angle of the structure in the closed position.

Depending on the number of structural elements (n), the values of X1 and X2 and their relative change (i.e., the value of expansion when stretched) are determined.

In this way, it is practically possible to calculate the theoretical values of the size of the planar structure.

**Figure 6:**
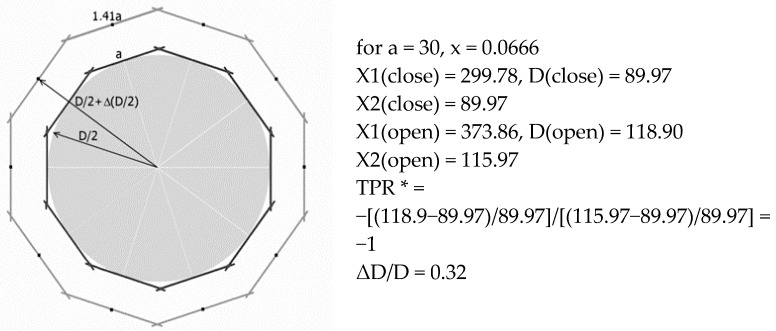
Schematic representation of a tubular structure built of 10 square frames times three rows in the closed position and in the open position, * Tube Poisson’s Ratio (TPR *) (with calculations).

If the above structure is rolled up (Figure 5), one can obtain an object in the form of a closed decagon in the projection on the base (Figure 6). The projection of the tubular structure shown forms a circle around which square frames are positioned. The change in the diameter of the circle corresponds to the expansion of the tubular structure in the direction perpendicular to the axis. In this regular and symmetrical arrangement of unit cells in the open position and in the closed position, a large radial expansion is evident, with both the diameter (as shown in the figure) and height increasing as a result of stretching.

The square frames touch the circle line of the tubular structure with a D/2 radius. It can be seen that the D diameter of the tubular structure is dependent on the size of the planar structures X1 and X2 since, according to the relationship:

X1 = πD(2)

while its height is X2 = H.

For the assumed parameters: a (length of the side of the square frame) and x (distance of the axis of rotation from the edge of the frame), the dimensions of the tubular structure were calculated, i.e., the diameter of the structure D and its height. As expected, a negative TPR = −1 and a diameter expansion of 32% were obtained. The change in the height of a tubular structure is due to axial displacement along its length, while the change in the diameter is due to radial expansion or contraction.

Therefore, changes in the size of the tubular structure correspond to changes in the size of the planar structure between the open and closed positions. The lateral and longitudinal expansion of the structure occurs in response to stretching and, conversely, in compression of an open structure, contraction is observed.

The above analysis indicates that the change in the size of the tubular structure will be of the same nature as the change in the size of the planar structure. Again, these changes can be determined as a function of the parameter x (that is, as a function of the position of the axis of rotation on the surface of the square frames).

**Figure 7:**
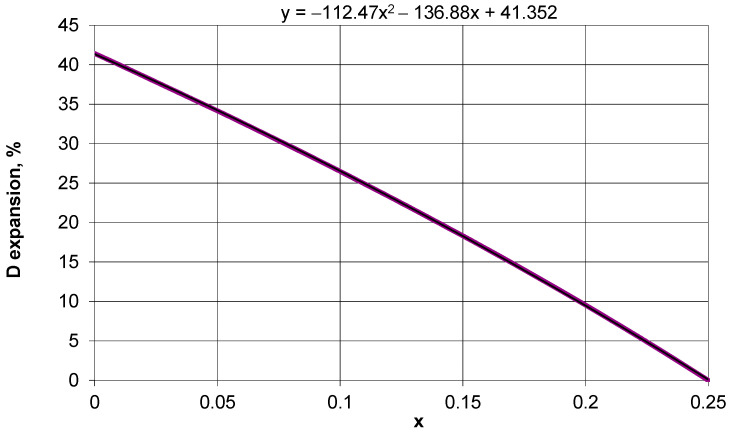
Expansion of the tubular structure along the diameter moving from closed to the open position.

Of particular interest is the possible expansion of the tubular structure when moving from closed to open positions. It is clear that the radius of the D/2 structure increases with the degree of opening. Square frames connected by rotation axes rotate and move to create an enlarged spatial openwork structure. The results of the calculations made for the above structure for different values of the parameter x in the coordinates: relative change in diameter of the tubular structure and parameter x are shown in Figure 7.

It is clear that the value of the parameter x is limited by the thickness t of the square frame. On the other hand, if there are full squares used instead of square frames, then the parameter x is in the range 0 < x < 0.25.

The obtained relationship between the expansion of the structure along the diameter and the parameter x is close to a straight line and shows that the closer the edge of the square frame is to the axis of rotation, the greater the expansion of the tubular structure that can be obtained. It is noteworthy that the maximum value of the expansion along the diameter of the tubular structure reaches a value of 41.42% for the parameter x = 0. For parameter x = 0.25, the squares overlap to the point where no rotation is possible, and therefore the expansion of the structure is equal to zero.

The relationship between the linear expansion of the structure as a function of the parameter x (shown in Figure 7) is a general relationship since it does not depend on the unit cell count of the structure or on the squared unit cell sizes.

Note that when it is stretched, the presented auxetic structure can undergo linear expansion within a wide range, e.g., for x = 0.125 (θ/2 = 10°), and the expansion is 22.5%, whereas most of the auxetic structures analyzed so far exhibit much lower expansion.

In turn, considering the ratio of the relative size change of the tubular structure along the diameter and along with the height, one can calculate the Poisson’s ratio as TPR, which is predicted to be −1.

**Figure 8:**
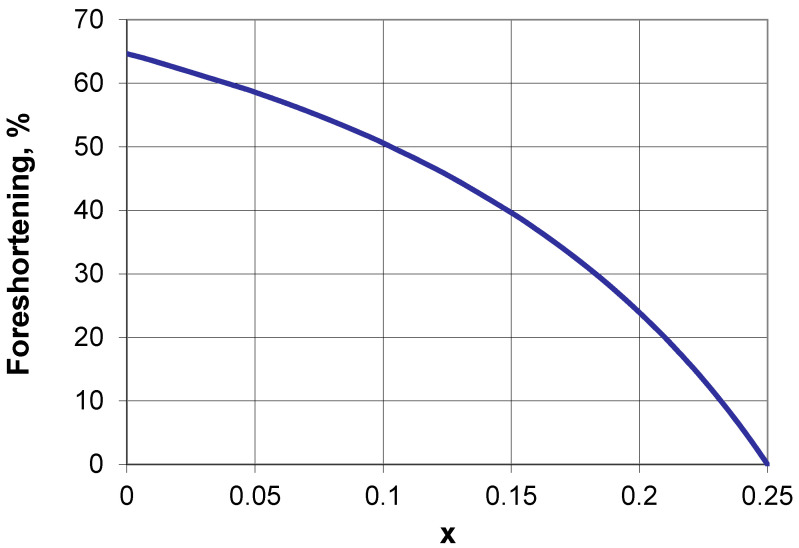
Shrinkage of the tubular structure, after the transition from open to the closed position, as a function of parameter x.

It is also interesting to see how the tubular structure shrinks if it is axially compressed from the open position to the closed position. Figure 8 shows the percentage of change in volume of the tubular structure when moving from the open position to the closed position. It became evident that due to axial shortening and radial contraction, the tubular structure shrinks (i.e., foreshortening).

Again, this relationship is only a function of the geometric parameter x and does not depend on either the size of the structure or the number of interconnected unit cells. As one can read from this graph, for small values of the x parameter (axis of rotation near the edge of the square), there are large values of foreshortening.

The results of experimental tests of the analyzed model will be presented later. A number of geometric configurations will be considered, and each of these cases can be realized in different ways, with the components of the structure-unit cells having a square form. This prevalent geometry has proven to have abundant implementation opportunities.

### 2.3. Planar Physical Structures

As already stated, structures built from unit cells in the form of square frames can take a variety of forms, although in any case, they can be given the shape typical of the initial structure of the rotating squares model. Different variants of the realization of structures from square units that satisfy the conditions of the square frames at a certain position are tested below.

**Figure 9:**
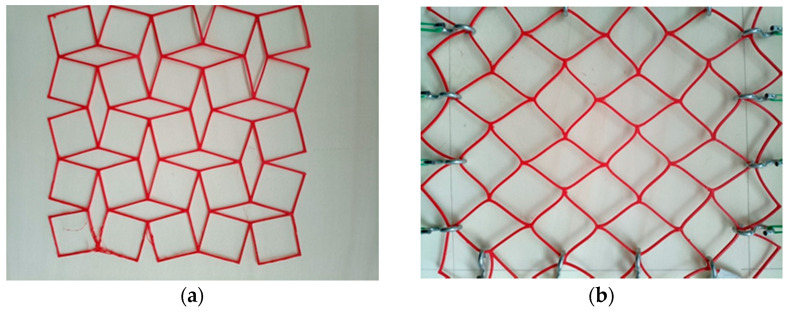
“Mesh” structure made of soft polymer in initial position (**a**) and after stretching in two perpendicular directions (**b**).

First, we present a design made by 3D printing, using filaments that allow for flexible construction. Figure 9 shows a structure realized from non-rigid squares resembling a fishing net. It can be arranged in a position resembling a model of rotating square frames, but to obtain both transverse and longitudinal expansion in stretching, it is necessary to apply force in two perpendicular directions. Such a “four-handed” action does not indicate an auxetic behavior of the structure in the form of a network.

The resulting deformation of this structure occurs mainly due to the high elastic properties of the material, with the individual non-rigid parts of the cells yielding in the direction of the tensile force.

**Figure 10:**
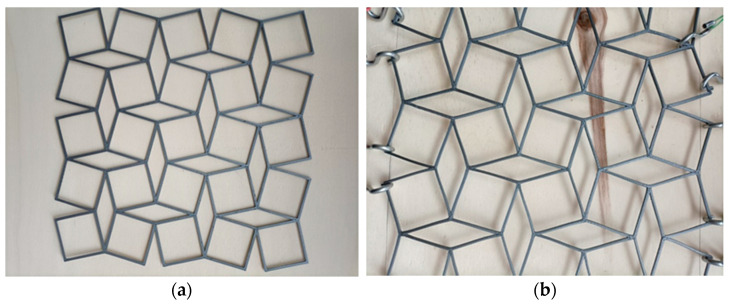
“Mesh” structure made of hard polymer, in initial position (**a**) and after stretching in two perpendicular directions (**b**).

In the next attempt, a hard polymer material was chosen for the 3D printing (Figure 10). In this case, due to low elasticity, the structure obtained only small deformations in stretching. Furthermore, it was found that the structure did not exhibit a hinge mechanism, and therefore only “four-hand” deformations could be realized with significant tensile forces.

These two types of experiments showed that the choice of material for the structure is as important as the structure itself.

**Figure 11:**
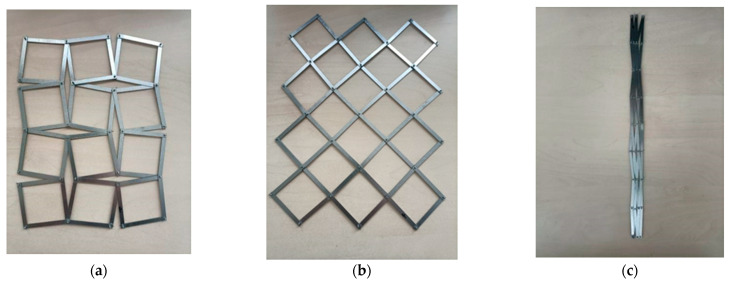
A structure made of metal bars connected to each other by axes of rotation at different stages of folding; pseudo-construction of rotating squares in the closed position (**a**) and in the open position (**b**) and folded state (**c**).

The third experiment involved a structure made of rigid elements connected on their surface by axes of rotation forming square frames. Figure 11 shows several possibilities for the arrangement of these elements, both forming rotating square-like structures and forms that are significantly different from them. In this design, there was hinging both between the square cells and within the cells themselves. In this case, there were many possibilities for the movement of the structure’s components.

This type of construction may also give the impression that it allows for the realization of an auxetic structure. In fact, as a result of the hinge, there are many possibilities for realizing various metamaterial constructions in this case. Unfortunately, however, neither of them is an auxetic structure.

This time, it was possible to see that the use of rigid elements which are hinged to form unit cells is not sufficient to realize an auxetic structure. Another test used rigid unit cells that were hinged together.

Another test used whole rigid unit cells that were hinged together. Such a use of rigid unit cells in the form of square frames, connected to each other in a novel way (namely by axes of rotation on their surface), makes it possible to achieve an excellent auxetic effect with a Poisson value of −1.

**Figure 12:**
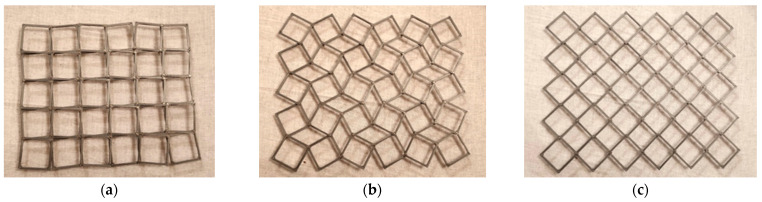
A 5 × 6 auxetic structure built with square frames connected by rotation axes on their surface in three positions *closed* (**a**), *partially open* (**b**) and *open* (**c**).

3D-printed square frames made of hard polymer (Figure 12), with holes for the rotation axes, can be assembled to form an auxetic structure, whereby the connections of the frames can be realized (e.g., by means of pins). The study of such a truss-like structure consisted in determining the change of dimensions X1 and X2 of the outline of the structure and in confirming the possibility of performing multiple cyclic changes of its size between positions conventionally assumed to be *open* and *closed*.

The rigid square frames presented can undergo rotation on the mounted rotation axes. When the structure is stretched in the longitudinal direction, it expands without failure, even in the transverse direction, and remains stable regardless of the rate of expansion and the number of cycles of tension and compression.

When such structures are stretched or compressed, the square units not only rotate, and the angles between the sides of adjacent squares change, but the squares move as well. This leads to planar expansion or contraction, which is a type of auxetic behavior. These auxetic reactions recognized by the geometrical analysis were each time confirmed experimentally.

The functional physical model of an auxetic planar structure obtained in this way can be a basis for the construction of a tubular structure.

### 2.4. Tubular Physical Structures

On the basis of the geometrical analysis presented in Section 2.1, tubular structures are feasible if they are rolled up to form planar structures. Taking into account that planar structures are made of movable unit cells and that the connections on rotary axes have some play, it is possible to roll them quite easily. The elastic properties possessed by unit cell materials can facilitate this operation, too.

**Figure 13:**
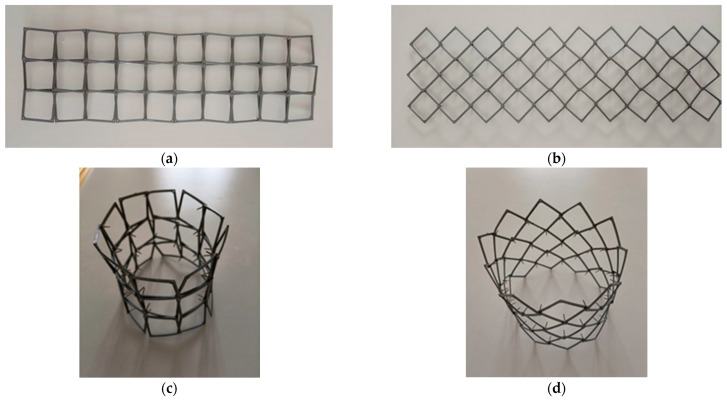
Planar structure of 10 × 3 square hard polymer frames (**a**,**b**) and a tubular structure after they are connected (**c**,**d**) in the closed and open positions.

Another test used a structure formed by square frames produced from a hard polymer using a 3D printing technique (Figure 13). The square frames were connected with rotation axes at their edges, thanks to which the structure could be produced. The square frames used exhibited high rigidity and poor flexibility.

Results of measurements of geometrical sizes of the structures are presented in Table 2.

The observed deformation of the short tubular structure involved significant deviations of the diameter at the upper end of the structure (96–104 mm).

From the resulting linear measurements, the Poisson’s ratio can be determined as the ratio of the expansion along the radius to the expansion along the axis of the tubular structure. The theoretical values given correspond exactly to an NPR of −1 since the theoretical relative linear expansion in both directions is the same. However, the measured data may deviate from the theoretical data due to the specified play in the rotational axes. Nevertheless, for the solutions used—constructions of rotating squares with rotation axes—the Poisson’s ratio is −1. Due to the differences in expansion in the longitudinal and transverse directions shown in the examples, more negative NPR values are obtained, which could be called ANPR (apparent negative Poisson’s ratio).

**Figure 14:**
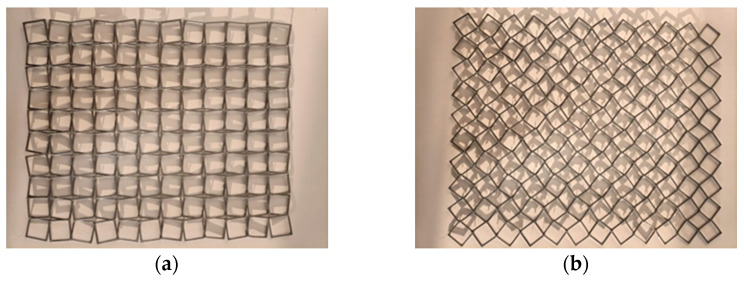

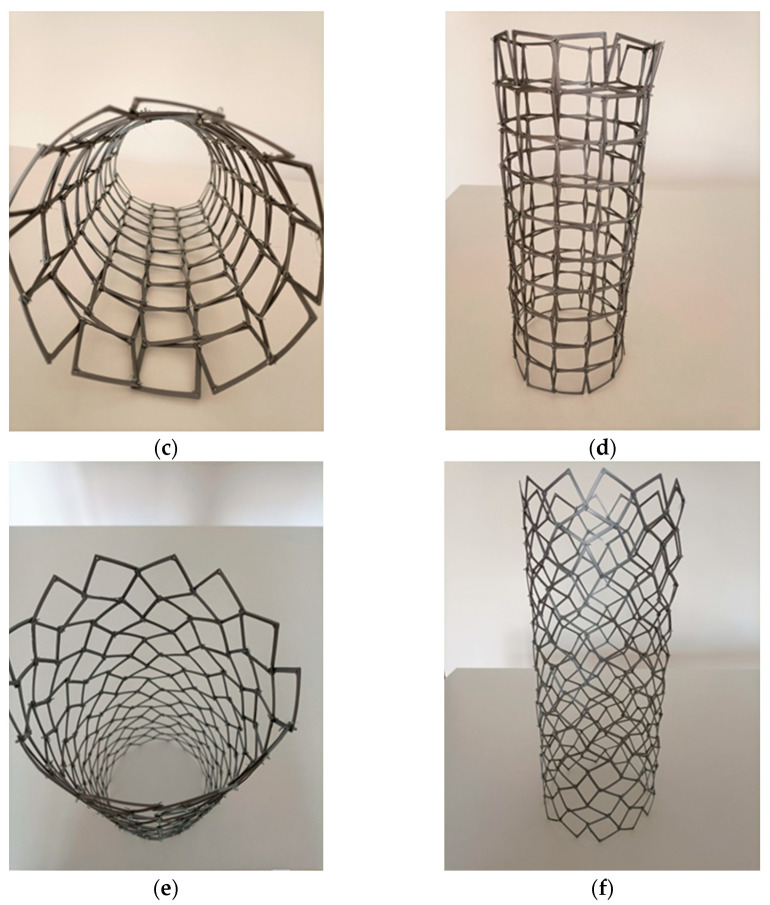
Expanded planar structure (**a**,**b**) of 12 × 10 square hard polymer frames and tubular structure (**c**–**f**) after joining in *closed* and *open* positions.

Expanding the above-described tubular structure to a 12 × 10 size (consisting of 12 × 10 repeating unit cells) resulted in the demonstration shown in Figure 14.

The experiment compared the initial planar structure and the tubular structure obtained from it after folding. The dimensions of the 12 × 10 structure in the presented variants are shown in Table 3.

In this case, for a design consisting of a large number of unit cells (120 units), a significant deviation of the experimental linear expansion value from the theoretical value is observed. The photographs (Figure 14) show that both the full opening and full closing of the structure were not achieved.

The reported values of size change obtained experimentally differ from the theoretical values for the parameter x = 0.072 in that the shrinkage determined experimentally was 49%, while the theoretical value was higher and reached 54%.

While the planar structure showed no resistance in tension and compression, the opposite was true for the tubular structure. The resistance to longitudinal stretching with expansion in the transverse direction increased with the number of square polymer frames incorporated into the structure. The tubular structure studied, due to the elastic properties and stiffness of the unit cell material, was largely resistant to buckling (lateral warping). In doing so, it exhibited an intrinsic deformation consisting of expansion at the ends of its tubular shape.

**Figure 15:**
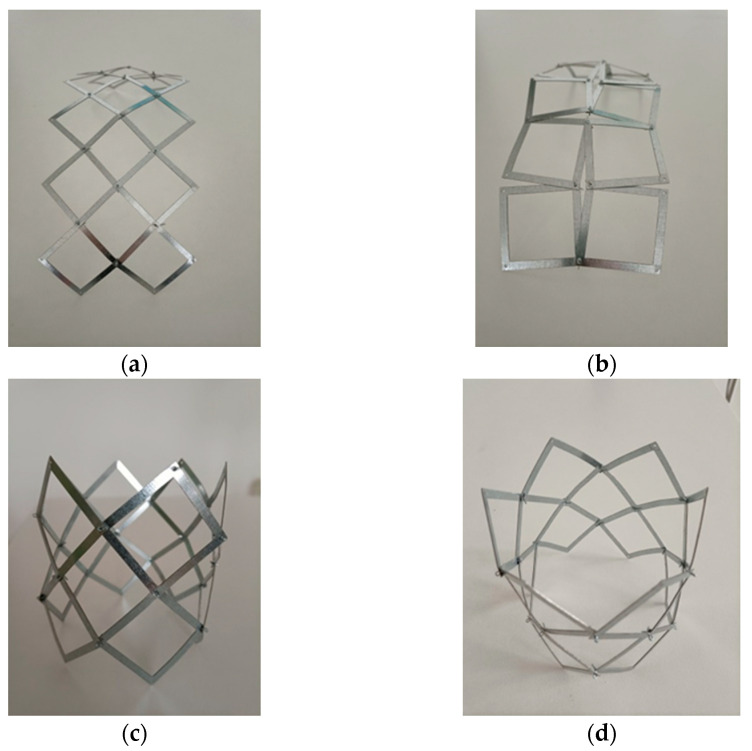
Planar construction of 6 × 2 square frames of a non-elastic material (**a**,**b**) and a tubular structure (**c**,**d**) after joining them, open positions (**c**) and closed positions (**d**).

Another such structure was made in another project of rigid metal frames, which, however, underwent a slight plastic deformation under the action of force. The photographs in Figure 15 illustrate that also these initially planar structures, after a slight bending, exhibit auxetic properties, and the transition from closed to open position can be realized for them through stretching.

However, the tubular structure obtained in this case showed a very high resistance when it was compressed and also when switching from the open to the closed position. While in the closed position, the structure expanded spontaneously at the ends; in the closed position, it showed a larger diameter in the middle.

## 3. Summary and Discussion

The present paper has attempted to verify the performance of metamaterial structures constructed from square unit cells by both geometrical analysis and by building physical models. The starting point was to determine the conditions for the operation of the hinge behavior (a mechanism) that for connected rigid square unit cells provides the basis for the auxetic properties. After checking the behavior of the structure in different material variants and ways of realization of square unit cells, it was confirmed that it is possible to build an auxetic structure by assembling a structure of square rigid frames connected with rotation axes on their edges.

By carefully arranging and connecting the rigid square frames, defects were avoided, and a replicable change in size was achieved in the transition from the *closed* to the *open* position. The rotation axis connections used avoided out-of-plane deformations and defects at the hinges. Although some unfavorable deformations appeared for short tubular structures, this effect was less clear for much larger structures.

By considering various possibilities for the realization of square cells and the construction of auxetic structures from them, the necessary conditions for this goal have been identified. It has been confirmed that it is convenient to use solid rigid squares or rigid square frames. For the realization of tubular structures, it is effective to use rigid elastic materials.

Thus, a broader class of metamaterial structures from the group of rotating squares was considered. What was achieved by changing the material realizations (e.g., the choice of material for unit cells) allowed us to focus on studying the performance of rigid square unit cells, connected by their rotation axes and on creating planar and tubular structures from them.

The position of the axis of rotation on a single square frame was determined by introducing a geometric parameter x, which is responsible for the angle of closing of the structure and its linear expansion. Depending on the value of the parameter x, which lies in the interval 0 < x < 0.25, the linear expansion lies in the range from 41.41% to zero and is, in this case, independent of the cell size and of the cell counts in the structure. In the same range, while, of course, depending on the parameter x, the diameter of the tubular structure can change. These values are incomparably larger than those considered in the auxetic structures analyzed [37,41,42,43,44,45].

By introducing rotation axes at the corners of the overlapping squares, we solved the problem of ensuring stable hinge connections, starting with experimental studies and then constructing a geometric model. The relationships found in this way provide a solid foundation for all such unit cell connections in auxetic structures. However, it must be stressed that the proposed rotary axis solution does not take advantage of the elastic properties of the unit cell material (i.e., the determined NPR is not related to these material modules).

It was also noted that there was no elastic deformation in the studied structures, but only interactions such as stress transfer and friction.

Thus, the entire tubular auxetic structure can undergo simultaneous stretching or shortening, in contrast to solutions in which there is a decreasing auxetic behavior from the center to both ends of the tubular structure (e.g., [44]). These specially designed and manufactured square frame connections allow for the most desirable hinges to withstand the specified load conditions.

In summary, this paper presents a novel concept for transforming a two-dimensional rotating squares model into a three-dimensional tubular structure that exhibits auxetic properties. While in the planar structures, their stretching and compression and thus the simultaneous change in size longitudinally and transversely occurred without resistance, the tubular objects constructed from these structures showed great resistance to their stretching and compression.

Considering the reliability of square unit cell connections with rotation axes, the performance of planar structures as well as tubular structures has been successfully assessed. It is particularly satisfactory that geometrical considerations can be fully utilized in experiments, and by setting the position of the axis of rotation in a single square unit cell, it is possible to determine the maximum elongation of the structure in tension.

The comparison of the physical tests of the auxetic structure with analytical results confirmed that the modified rotating square frame structure has been adequately understood. The materials for the unit cells were selected from two groups: rigid polymers and metal sheets. Connections of the unit cells to the rotation axes in the form of pins or thin screws were a temporary solution and could be replaced by (e.g., suitably sized tube rivets). This element, in the case of tubular structures, determines the accuracy of the connections and the nature of friction between the planes of the square frames. Therefore, future research should focus on finding more advantageous solutions to the hinge connection problem than pins.

## 4. Conclusions

The present work utilized a wide range of auxetic structures designed and built from unit cells based on Grim and Evans rotating squares. Different types of hierarchical geometry were used, from a surface covered by solid squares to an object in the form of a lattice. The designs considered were evaluated using geometrical analysis and experimentally created physical models. The results obtained showed that the application of geometry based on square frames leads to the same auxetic behavior as for full squares. Auxetic structures based on square frames can be ideal candidates for the design of lightweight systems.

The present paper also presents the possibility of realizing tubular auxetic structures, depending on the type of unit cell material used. The tensile properties of the structures were studied, and the experimentally determined changes in diameter and height of the structures were consistent with model calculations. The model was based on an analysis of the geometric relationships of the auxetic structure.

The experimentally generated physical models in the form of planar structures constructed from square frames and tubular structures made from them both exhibited auxetic properties. The transition from closed to open positions in the structures was associated with a large elongation of the structures and led to a Poisson’s ratio of −1.

In general, it can be concluded that the experimentally fabricated tubular structures showed proper and satisfactory performance in both types of acting force (i.e., in compression and tension).

The use of metamaterials and structures based on rotating square unit cells connected by rotation axes, especially in the form of tubular structures, may be of increasing interest since such structures can function in a very stable manner and provide large volume compression values.

## Figures and Tables

**Table 1:** Geometric relationships needed to calculate the size of the structure in the closed position and in the open position (see Figure 5).

Position	c	d	e	f
*close*	$a\;\times \;\sin\frac{\theta }{2}$	$\frac{a\times \cos\frac{\theta }{2}}{1+\tan\frac{\theta }{2}}$	$a\;\times \;\cos\frac{\theta }{2}$	$2a\frac{\tan\frac{\theta }{2}\sin\frac{\theta }{2}}{1+\tan\frac{\theta }{2}}$
*open*	$\frac{a}{\sqrt{2}}$	$\frac{a\times \left(1-2x\right)}{\sqrt{2}}$	---	---

**Table 2:** Comparison of the measured and theoretical values for the planar structure and the resulting tubular structure.

Structure: 10 × 3, for a = 32 mm, x = 0.0625 (Theoretical Expansion 32.29%)
**Planar**
experimental: X1(open) = 404 mm, X1(close) = 302 mm, expansion 33.77% X2(open) = 123 mm, X2(close) = 92 mm, expansion 33.69%
**Tubular**
experimental: D(open) * = 119 mm, D(close) * = 96 mm, expansion 23.95% X2(open) = 123 mm, X2(close) = 90 mm, expansion 36.66%

* deformation.

**Table 3:** Comparison of the measured and theoretical values for the planar structure and the resulting tubular structure.

Structure: 12 × 10, for a = 32 mm, x = 0.072 (Theoretical Expansion 30.04%)
**Planar**
experimental: X1(open) = 472 mm, X1(close) = 377 mm, expansion 25.19% X2(open) = 397 mm, X2(close) = 317 mm, expansion 25.23%
**Tubular**
experimental: X1(open) = 471 mm, X1(close) = 377 mm, expansion 24.93% D(open) = 150 mm, D(close) = 120 mm, expansion 25.01%

## Data Availability

All data have contained within the article.
